# Supplementary material for: Exact invariant solution reveals the origin of self-organized oblique turbulent-laminar stripes
Source: Nat Commun. 2019 May 23;10:2277. doi: 10.1038/s41467-019-10208-x (PMC6533313; doi:10.1038/s41467-019-10208-x)
Supplement: Supplementary file 2 — Description of Additional Supplementary Files [file 41467_2019_10208_MOESM2_ESM.pdf]

Description of Additional Supplementary Files

Supplementary Data 1

The fully resolved velocity field of the stripe equilibrium is provided as a data file in the supplementary material. A description of the NetCDF file, including grid variables and how to reproduce the equilibrium using the open source software CHANNELFLOW, is given in the section `data availability`.
